# Plasma progesterone profile and conception rate following exogenous supplementation of gonadotropin-releasing hormone, human chorionic gonadotropin, and progesterone releasing intra-vaginal device in repeat-breeder crossbred cows

**DOI:** 10.14202/vetworld.2016.559-562

**Published:** 2016-06-05

**Authors:** N. K. J. Pandey, H. P. Gupta, Shiv Prasad, S. K. Sheetal

**Affiliations:** Department of Veterinary Gynaecology and Obstetrics, College of Veterinary & Animal Sciences, G.B. Pant University of Agriculture and Technology, Pantnagar - 263145, Uttarakhand, India

**Keywords:** human chorionic gonadotropin, gonadotropin-releasing hormone, progesterone impregnated device, repeat breeding

## Abstract

**Aim:**

This study was designed to evaluate the effect of gonadotropin-releasing hormone (GnRH), human chorionic gonadotropin (hCG), and progesterone impregnated intra-vaginal device on progesterone profile and conception rate in repeat-breeding crossbred cows.

**Materials and Methods:**

Repeat-breeding crossbred cows aged 3-8 years (n=32), lactating and negative to white side test were randomly divided into four groups: Group 1 (Control, n=8), Group 2 (GnRH at 10 µg i.m, n=8), Group 3 (hCG at 1500 IU i.m., n=8), and Group 4 (progesterone impregnated intra-vaginal device at 958 mg, n=8). All the treatme nts were given on 5^th^ daypostbreeding and in Group 4 intra-vaginally implanted device was withdrawn on 9^th^ day (i.e., implant inserted for total 4 days) of the estrous cycle. Blood samples were collected on day 0, 5, 10, 15, and day 20 of estrous cycle, and plasma was separated for progesterone estimation.

**Results:**

Accessory corpus luteum was not formed in crossbred cows of Group4 and control group. However, total 6 and 8 accessory corpora lutea were found in Group 2 and Group 3, respectively. In pregnant cows, the plasma progesterone concentration increased continuously from day 0 to day 20. In non-pregnant cows, it increased from day 0 to day 15 and then declined. The conception rate on day 60 in Group 1, Group 2, Group 3, and Group 4 was 37.5%, 50%, 75%, and 37.5%, respectively.

**Conclusions:**

Treating repeat-breeder cows with hCG is effective in increasing conception rate by developing accessory corpora lutea and higher progesterone level.

## Introduction

Over the past five decades, the milk production increased while the pregnancy rate in dairy cows rapidly decreased [1] due to changes in reproductive physiology [2], resulting in increased number of services per conception [3]. Consequently, the incidence of repeat breeding might have increased [4] and had very poor reproductive performance, despite repeated insemination [5]. The repeat-breeding cow is one that has normal or nearly normal estrous cycles and estrous periods and has been bred 2 or more times to a fertile bull yet failed to conceive. The clinical examination of the animal may fail to reveal any definite lesion or condition to explain the failure of conception [6].

Embryonic mortality is one of the predominant causes for repeat breeding in dairy animals [7]. The majority of embryonic mortality (70-80% of total loss) occurs between days 8 and 16 after insemination [8]. Luteal insufficiency is a known cause of it. Luteal deficiency during the very early stages of pregnancy has been hypothesized as a cause of pregnancy failures [9]. Low plasma progesterone may affect reproductive process before and after insemination. A delayed progesterone rise and lower total progesterone concentrations are the most prominent endocrine deviations in repeat-breeding cow [9]. On day 16, cows with high progesterone concentrations have larger embryos than those of cows with lower progesterone levels. Progesterone is essential for orchestrating the histotrophic environment for the nourishment of the conceptus [8].

During the pre-implantation phase of embryonic development, direct progesterone supplementations [10] and gonadotropin-releasing hormone (GnRH)/human chorionic gonadotropin [11,12] injections are used to improve embryonic survival in repeat-breeder cows.

The objective of this study was to determine the effects of GnRH, human chorionic gonadotropin (hCG), and progesterone impregnated device on progesterone profile and conception rate in repeat-breeding crossbred cows.

## Materials and Methods

### Ethical approval

The present investigation was carried out after the approval of the Institutional Animal Ethics Committee.

### Selection and maintenance of the animals

The study was conducted on 32 repeat-breeding crossbred cows aged 3-8 years at Instructional Dairy Farm, Nagla, G. B. Pant University of Agriculture and Technology, Pantnagar- 263 145, District Udham Singh Nagar (Uttarakhand) from January to April 2015. All animals were thoroughly examined per-rectally to rule out any anatomical defect of genitalia and ovarian abnormalities. They were kept under uniform feeding and managemental conditions. Cows included in the study were in lactation phase having lactation yield between 3000 and 3500 L, negative to white side test and had more than two unsuccessful inseminations within the current lactation. Cows were inseminated twice (12 h interval) at normal estrus. We hypothesized that supplementation of exogenous progesterone or administration of GnRH or hCG during early luteal phase could overcome the luteal insufficiency and increase the conception rate in repeat-breeding crossbred cows. Whiteside negative animals were randomly divided into four groups (each group containing 8 animals): Group1 (Control, n=8), Group 2 (buserelin acetateat 10 µg i.m., n=8), Group 3 (hCG at 1500 IU i.m., n=8), Group 4 (progesterone impregnated intra-vaginal device at 958 mg, n=8). All the treatments were given on 5^th^ daypostbreeding, and in Group 4, intra-vaginally implanted device was withdrawn on 9^th^ day (i.e., implant inserted for total 4 days) of estrous cycle postbreeding.

### Collection of blood samples, progesterone estimation, and pregnancy diagnosis

The plasma samples from the blood of all the animals were collected during hormonal treatment on 0, 5, 10, 15, and 20^th^ day. The area of blood collection site was cleaned and disinfected with the spirit cotton swab to minimize the contamination. The blood was collected aseptically from jugular vein with the help of sterilized disposable syringe and 18G needles in ethylenediaminetetraacetic acid vials. After collection, the blood samples were brought to the laboratory in thermocol box containing ice packs. Plasma was separated by centrifuging blood samples at 3000 rpm for 15 min. Then, plasma samples were transferred into sterilized vials and stored at −20°C for progesterone estimation. Progesterone estimation was done using Progesterone C.T. RIA kit (M/S Beckman Coulter IM 1188) at IVRI (Division of Physiology and Climatology). Pregnancy was confirmed through per rectal examination and ultrasonography on 60 days post-artificial insemination (AI) using rectal transducer of 5.0 MHz frequency.

### Statistical analysis

The data were analyzed statistically using analysis of variance [13].

## Results and Discussion

The mean plasma progesterone concentration (ng/ml) in pregnant cows of Groups 1, 2, 3, and 4 on day 5 of pregnancy was 1.60±0.59, 1.41±0.09, 1.70±0.46, and 1.80±0.23, respectively. However, there was a significant increase in plasma progesterone concentration between day 0 and 5 in all groups, but no significant difference was observed on day 5 between the groups. The plasma progesterone concentration of Groups 2, 3, and 4 was higher in comparison to control group on day 10 but only Groups 3 and 4 have significantly (p<0.05) higher progesterone concentration than control (Table-1). The plasma progesterone concentration of Groups2 and 3 was significantly (p<0.05) higher than pregnant animal of control on day 15 and 20 (Table-1). This finding is showing the effect of hormonal treatment in treated groups. The plasma progesterone concentration of non-pregnant animal (within group) was significantly higher than the control group on day 10 (Table-2). Non-pregnant animal of the treated group did not differ significantly with the control group on day 15 and 20 (Table-2). In non-pregnant animal of all the groups, the plasma progesterone concentration has increased up to day 15 and then decreased due to luteolysis of corpus luteum (CL). On day 15, pregnant animals of all groups have higher progesterone concentration as compared to non-pregnant animal of the same group (Tables-1 and 2). It is in agreement with the finding of Patel *et al*. [14], who reported that level of plasma progesterone steadily increased from day 0 of the estrous cycle, attained peak level on day 14 and started declining thereafter in non-pregnant animals. Pandey *et al*. [15] also reported the significantly higher level of plasma progesterone in pregnant buffaloes (p<0.05) than in non-pregnant buffaloes.

**Table-1:** Comparison of mean plasma progesterone values of pregnant animals of different groups on different days of the estrous cycle.

Treatment/days	Group 1 (Control) (n=3)	Group 2 Buserelin acetate at 10 µg i.m., on 5^th^ day post-AI (n=4)	Group 3 hCGat 1500 IU i.m., on 5^th^ day post-AI (n=6)	Group 4 Progesterone impregnated intra-vaginal device on 5^th^ day post-AI withdrawn on 9^th^ day (n=3)
Day 0	0.17^Ac^±0.02	0.23^ABd^±0.05	0.38^Bd^±0.05	0.37^Bc^±0.08
Day 5	1.60^b^±0.59	1.41^d^±0.09	1.70^d^±0.46	1.80^c^±0.23
Day 10	3.58^Ba^±0.62	4.22^ABc^±0.48	5.19^Ac^±0.92	6.85^Ab^±1.37
Day 15	4.54^Ba^±0.29	7.77^bA^±1.41	7.67^bA^±0.76	4.42^aB^±0.21
Day 20	4.76^Ba^±0.38	10.24^Aa^±1.03	10.68^Aa^±0.69	4.60^Ba^±0.57

Means bearing different superscripts differed significantly (p<0.05) within the groups (^a, b, c, d^) and between the groups (^A, B^). AI=Artificial insemination

**Table-2:** Study of mean plasma progesterone values of non-pregnant animals of different groups on different days of the estrous cycle.

Treatment/days	Group 1 (Control) (n=5)	Group 2 Buserelin acetate at 10 µgi.m., on 5^th^ day post-AI (n=4)	Group 3 hCG at 1500 IU i.m., on 5^th^ day post-AI (n=2)	Group 4 Progesterone impregnated intravaginal device on 5^th^ day post-AI withdrawn on 9^th^ day (n=5)
Day 0	0.26^c^±0.06	0.23^b^±0.09	0.12^c^±0.06	0.41^c^±0.12
Day 5	1.42^abc^±0.55	1.33^b^±0.33	0.77^c^±0.14	1.30^c^±0.08
Day 10	2.50^Bab^±0.87	4.13^Aa^±1.06	2.62^Bb^±0.17	5.77^Aa^±0.71
Day 15	3.31^a^±0.94	4.34^a^±1.70	4.95^a^±0.99	3.99^b^±0.33
Day 20	0.62^bc^±0.28	0.99^b^±0.31	0.62^c^±0.13	0.39^c^±0.10

Means bearing different superscripts differed significantly (p<0.05) within the groups (^a, b, c^) and between the groups (^A, B^). AI=Artificial insemination

In Groups 2 and 3, higher progesterone concentration is due to formation of accessory CL from dominant follicle of the first follicular wave. In Groups 2 and 3, the presence of accessory CL confirmed through per-rectal examination on day 15. Maximum first service conception rate was observed in Group 3 (75%) and Group 2(50%) in comparison to control group (37.5%) (Table-3). There is a positive effect of induced accessory CL on the conception rate [16]. Our finding was in agreement with the observations of many workers [12,17] who reported that administration of hCG/GnRH in early luteal phase between day 4 and 7 of the estrous cycle induced ovulation of the first wave dominant follicle and formation of accessory CL. The accessory CL increases progesterone concentration and could decrease estrogenic environment during the period of pregnancy recognition. This increased level of progesterone has a positive effect on embryo survival. Buserelin acetate improved conception in cows if administered either on day 0 along with AI or day 12 post insemination [11]. This is also in agreement with the findings of Musilová *et al*. [18], who reported that administration of GnRH on day 5-7 or 11-13 after insemination was efficient to induce accessory CL and increase the concentration of serum progesterone. Several studies have reported a positive effect of GnRH administration on pregnancy rates [19-21], but in few other studies, GnRH administration failed to exhibit a positive impact on pregnancy rate [22, 23].

**Table-3:** Conception rate.

Groups	Group 1	Group 2	Group 3	Group 4
Total number of cows	8	8	8	8
Total number of pregnant cows	3	4	6	3
First service conception rate (%)	37.50	50	75	37.50

In pregnant animal of Group 4, the plasma progesterone concentration was significantly (p<0.05)was reduced between day 10 and 15. On day 15, there was no significant difference observed between Group 4 and control (Table-1). In Group 4, due to removal of intra-vaginal implant, mean plasma progesterone concentration has decreased. In Group 4, conception rate was same as control group (37.5%). This finding is in agreement with the finding of Carothers [24], who reported that providing supplemental progesterone after breeding for 7 days or 14 days to heat-stressed dairy cows did not increase serum progesterone concentration. The above finding is in agreement with the findings of Rathbone *et al*. [25], who demonstrated with ovariectomized Holstein cows that serum progesterone concentrations begin to rise within 1 h following controlled internal drug release (CIDR) insertion, elevating the concentration to levels normally seen during the luteal phase of the estrous cycle; and progesterone concentrations begin to decrease within 1 h after CIDR removal. Inserting the progesterone impregnated device on the 5^th^ day after insemination gives the CL approximately 5 days to develop, meaning that the CL should be fully formed and secreting progesterone for a normal lifespan. It is reported that progesterone supplementation may not translate into improved pregnancy rates possibly due to its potentially negative effects on CL lifespan [26].

## Conclusions

Application of hCG and GnRH on 5^th^ day postbreeding improved conception rate and also increased the plasma progesterone concentration before critical period of maternal recognition of pregnancy. However, direct progesterone supplementation for a short period, i.e., between days 5 and 9 did not improve conception rate. Progesterone profile was higher during supplementation of progesterone but after removal of exogenous source progesterone concentration started declining.

## Authors’ Contributions

NKJP carried out the experiment and drafted the final manuscript. HPG designed the experiment, guided during the experiment. SP and SKS helped in the analysis of the data and scientifically corrected the manuscript. All authors read and approved the final manuscript.
